# A facile method for expression and purification of the Alzheimer’s disease-associated amyloid β-peptide

**DOI:** 10.1111/j.1742-4658.2008.06862.x

**Published:** 2009-03

**Authors:** Dominic M Walsh, Eva Thulin, Aedín M Minogue, Niklas Gustavsson, Eric Pang, David B Teplow, Sara Linse

**Affiliations:** 1 Laboratory for Neurodegenerative Research, School of Biomolecular and Biomedical Science, Conway Institute, Belfield, University College DublinRepublic of Ireland; 2 Department of Biophysical Chemistry, Chemical Centre, Lund UniversitySweden; 3 Department of Biochemistry, Chemical Centre, Lund UniversitySweden; 4 Biopolymer Laboratory, David Geffen School of Medicine at UCLALos Angeles, CA, USA

**Keywords:** Aß, Alzheimer's disease, aggregation, amyloid, fibrillogenesis

## Abstract

We report the development of a high-level bacterial expression system for the Alzheimer’s disease-associated amyloid β-peptide (Aβ), together with a scaleable and inexpensive purification procedure. Aβ(1–40) and Aβ(1–42) coding sequences together with added ATG codons were cloned directly into a Pet vector to facilitate production of Met-Aβ(1–40) and Met-Aβ(1–42), referred to as Aβ(Μ1–40) and Aβ(Μ1–42), respectively. The expression sequences were designed using codons preferred by *Escherichia coli*, and the two peptides were expressed in this host in inclusion bodies. Peptides were purified from inclusion bodies using a combination of anion-exchange chromatography and centrifugal filtration. The method described requires little specialized equipment and provides a facile and inexpensive procedure for production of large amounts of very pure Aβ peptides. Recombinant peptides generated using this protocol produced amyloid fibrils that were indistinguishable from those formed by chemically synthesized Aβ1–40 and Aβ1–42. Formation of fibrils by all peptides was concentration-dependent, and exhibited kinetics typical of a nucleation-dependent polymerization reaction. Recombinant and synthetic peptides exhibited a similar toxic effect on hippocampal neurons, with acute treatment causing inhibition of MTT reduction, and chronic treatment resulting in neuritic degeneration and cell loss.

Multiple lines of evidence indicate that the amyloid β peptide (Aβ) plays an important role in the pathogenesis of Alzheimer’s disease [1]. In nature, Aβ does not occur as a single molecular species, and more than 20 different Aβ sequences have been detected in human cerebrospinal fluid and brain. The most common Aβ isoform is Aβ1–40, a 40-residue peptide that begins at Asp1 and terminates at Val40 (Fig. 1) [2–11]. Increased production of Aβ1–42, a peptide that differs from Aβ1–40 by addition of Ile and Ala to the C-terminus, is particularly associated with disease [12]. Through biochemical and animal modeling studies, researchers have built up a detailed picture of the natural economy of brain Aβ. Like all proteins, the steady-state level of Aβ is controlled by its production, degradation and clearance, and it is proposed that a defect leading to over-production or decreased clearance causes an accumulation of Aβ and that this triggers a pathogenic cascade culminating in the cognitive deficits that characterize Alzheimer’s disease [13–16]. The self-association constants of Aβ are relatively high, and a variety of assemblies are formed at micromolar concentrations, ranging from dimers to aggregates of amyloid fibrils [17]. However, as yet the specific form(s) of Aβ that causes injury to neurons *in vivo* has not been identified [16]. Clearly a detailed understanding of the structure of both the Aβ monomer and its various assemblies could help in the design of new therapeutic strategies targeted at preventing the formation or ameliorating the activity of toxic Aβ assemblies.

**Fig. 1:**
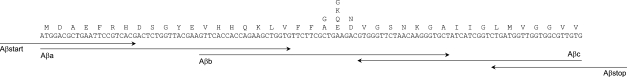
Aβ primary sequence and primers used to construct an Aβ synthetic gene. The amino acid sequence of Aβ(M1–40) is shown, with the disease-associated amino acid substitutions indicated above the residues that are replaced. The *E. coli*-optimized DNA sequence shown below the corresponding amino acids, and the primers used to generate the synthetic gene are indicated by arrows (full sequences are given in Experimental procedures).

Although much progress has been made since the sequence of Aβ was first determined, high-resolution structural analysis of Aβ monomer and its assemblies has been hampered because of the lack of an affordable source of Aβ peptides. Chemical synthesis of various Aβ peptides is now routine [18,19], but is time-consuming and requires access to specialized equipment, and is relatively expensive, especially for isotope labeling. Moreover, solid-phase synthesis of Aβ peptides containing radioisotopes such as ^35^S-Met is not practical. Thus we aimed to develop a simple inexpensive procedure for the production of recombinant Aβ peptides that would allow isotope labeling and the generation of Aβ peptides with design or disease-associated amino acid substitutions. Production and purification of recombinant Aβ peptides has been investigated previously, but most published methods either require highly specialized equipment and/or expensive reagents [20–22], or are only suitable for the production of short biologically irrelevant fragments of Aβ [23]. Here we describe a rapid and inexpensive protocol for the expression and purification of Aβ(1–40) and Aβ(1–42) with exogenous initiating Met residues. This procedure does not require specialized equipment, is suitable for isotopic labeling of peptides, and can be readily adapted for the generation of Aβ peptides containing an array of sequence variations.

## Results

### Expression of Aβ(M1–40) and Aβ(M1–42)

Sequence-verified PetSac plasmids containing either the Aβ(Μ1–40) or Aβ(Μ1–42) gene (Fig. 1) were used for expression in *Escherichia coli* as described in Experimental procedures. For Aβ(M1–40) and Aβ(M1–42), the highest yields were obtained between 3 and 4 h after induction, with similar yields at concentrations of isopropyl thio-β-d-galactoside ranging from 0.1–1.2 mm and temperatures ranging from 37–41 °C (data not shown). Under these conditions, the cells grow to an attenuance at 600 nm (*D*_660 nm_) of 3.0–3.1.

**Fig. 2:**
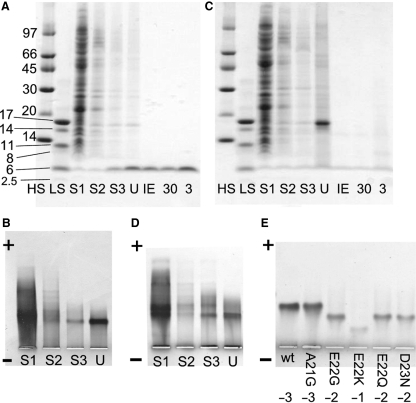
Aβ(M1–40) and Aβ(M1–42) are expressed in inclusion bodies. (A–D) Pellets of bacteria expressing Aβ(M1–40) (A,B) or Aβ(M1–42) (C,D) were subjected to three rounds of sonication in buffer, and at the end of each sonication step the suspension was centrifuged and the supernatants (labeled S1, S2 and S3) were stored pending analysis. The pellet was then extracted in 8 m urea (fraction labeled U), and purified by ion exchange (fraction labeled IE), filtration through a 30 kDa molecular mass cut-off filter (fraction labeled 30) and concentration on a 3 kDa molecular mass cut-off filter (fraction labeled 3). All fractions were electrophoresed on 10–20% polyacrylamide Tris-tricine gels (A,C) and 1% agarose gels (B,D), and proteins were visualized by Coomassie stain. Lanes HS and LS are molecular mass standards, with the molecular mass in kDa given on the left. (E) 1% agarose gel electrophoresis of urea extracts of inclusion bodies from bacteria expressing Aβ(M1–40) with wild-type (wt) sequence or with the following point mutations: A21G, E22G, E22K, E22Q and D23N. The net charge of each peptide is indicated underneath each lane.

SDS-PAGE and agarose gel electrophoresis of sonicates of the bacterial cell pellet and the urea extract revealed that the first and second supernatants after sonication contained mainly *E. coli* proteins, and the majority of Aβ(M1–40) and Aβ(M1–42) was present in the urea extract (Fig. 2). On agarose gels, the major band migrated as expected according to the net charge of the Aβ peptides at pH 8.4, and on SDS-PAGE the major band migrated between 4 and 5 kDa (Fig. 2). These data indicate that both peptides accumulate in inclusion bodies, and that Aβ(Μ1–40) is the dominant protein in the inclusion bodies. In contrast, the major protein in the Aβ(M1–42) inclusions was not Aβ, but was the small heat shock protein IbpB (accession number B1IYQ8), identified by mass spectrometry after tryptic digestion of the gel band (data not shown).

The PCR protocol used to generate Aβ(M1–40) and Aβ(M1–42) was designed to facilitate incorporation of familial mutants by exchange of only the middle primer. We produced six plasmids encoding Aβ(M1–40) that incorporate the point mutations F19P, A21G, E22G, E22K, E22Q and D23N, and another six plasmids encoding Aβ(M1–42) with the point mutations F19P, A21G, E22G, E22K, E22Q and D23N. These mutated versions can be expressed and purified using the procedure described here, although the higher aggregation tendency of some of these mutants leads to lower yields. On agarose gel electrophoresis, the peptides were found to migrate according to their respective net charge relative to wild-type (Fig. 2E).

### Purification of Aβ(M1–40) and Aβ(M1–42)

**Fig. 3:**
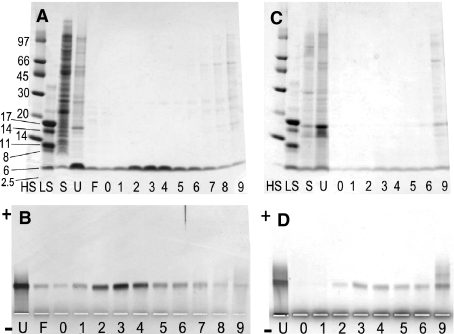
Ion-exchange purification of urea-solubilized inclusion bodies. Anion-exchange chromatography in batch mode was performed for Aβ(M1–40) (A,B) and Aβ(M1–42) (C,D). All fractions were electrophoresed on 10–20% polyacrylamide Tris-tricine gels (A,C) or 1% agarose gels (B,D), and proteins were visualized by Coomassie stain. S, combined supernatants after sonication and centrifugation; U, urea-solubilized pellet after third sonication; F, flow-through from application to ion-exchange resin. The peptides were eluted using a stepwise increase in NaCl concentration, and the fractions are labeled as follows: lane 0, 0 mm; lane 1, 50 mm; lane 2, 75 mm; lane 3, 100 mm; lane 4, 125 mm; lane 5, 150 mm; lane 6, 200 mm; lane 7, 250 mm; lane 8, 300 mm; lane 9, 500 mm NaCl. HS and LS, high and low molecular mass standards with the molecular mass in kDa given on the left.

The present work describes a rapid and inexpensive purification scheme to produce high-purity Aβ(M1–40) and Aβ(M1–42) in 24 h. The purification scheme, as described in detail in Experimental procedures, involves ion-exchange chromatography in batch mode, followed by molecular mass fractionation using centrifugal devices. This simple two-step purification results in a highly pure product, and yields 10–20 mg of Aβ(M1–40) per liter of culture. In the example shown in Fig. 3, 30 mg of peptide was obtained from 2.2 L of bacterial culture. The process can easily be scaled proportionally for other amounts. In the example shown in Fig. 3, the resin was washed with low-salt buffer followed by stepwise elution using 50, 75, 100, 125, 150, 200, 250, 300 and 500 mm NaCl, and fractions eluted using 50–125 mm NaCl were collected for molecular mass fractionation. In later batches, we washed the resin with buffer containing 25 mm NaCl and then eluted the peptide with buffer containing 125 mm NaCl, simplifying the procedures even further.

Urea-solubilized inclusion bodies containing Aβ(M1–42) were purified by anion-exchange chromatography in the same fashion as for Aβ(M1–40) (Fig. 3C,D). Fractions eluted with 75–125 mm NaCl were passed through a 30 kDa molecular mass cut-off filter, yielding a total of 5 mg of Aβ(M1–42) in 150 mL. Another 3 mg in 100 mL was obtained in the 30 kDa filtrate from fractions eluted at 150–200 mm NaCl. For both Aβ(M1–40) and Aβ(M1–42), all manipulations were performed at slightly alkaline pH to avoid the formation of structural contaminants produced by isoelectric precipitation. Depending on the required use, peptides can be lyophilized, used directly or concentrated.

### Ion-exchange column chromatography

Attempts to purify Aβ(M1–40) or Aβ(M1–42) by ion-exchange column chromatography (not shown) led to much lower yields of monomeric peptide than the batch method. When repeated using 8 m urea-containing buffers, the yields of eluted peptide were as high as or higher than with the batch mode, but the peptide was eluted at very high concentration and the majority of the material did not pass through the 30 kDa filter.

### Concentration of purified Aβ(M1–40) and Aβ(M1–42)

Aβ(M1–40) and Aβ(M1–42) each contain a single tyrosine residue, and absorption of tyrosine at 275 nm (ε_275_ = 1400 m^−1^·cm^−1^) was used to estimate the concentration of Aβ in solution. In four separate purification experiments, the concentration of Aβ(M1–40) in the 30 kDa filtrate was determined to be between 30 and 50 μm. The average Aβ(M1–40) concentration in the 30 kDa filtrate of the peak fractions (eluting at 75–125 mm NaCl) was 40 μm, based on the absorbance at 275 nm. This concentration is higher than required for thioflavin T (ThT)-based fibrillation assays (typical concentrations used are 3–10 μm), but is not sufficient for other biophysical studies. We therefore examined a number of methods to further concentrate the Aβ solution. Although several different methods proved useful (e.g. C18 SepPak reverse-phase columns), the best yield and most rapid results were obtained using a 3 kDa molecular mass cut-off centrifugal filtration device. When a solution of Aβ(M1–40) of approximately 40 μm was concentrated approximately eightfold, approximately 75% of the peptide was recovered at a concentration of approximately 230 μm.

### Amino acid analysis, mass spectrometry and sequencing

The purified peptides were subjected to mass spectrometry, amino acid analysis and N-terminal amino acid sequencing. These methods confirm expression of the correct peptide and that the peptide species contains the N-terminal methionine residue. For Aβ(M1–40), the observed relative molecular mass (mono-isotopic mass) was 4459.19 (expected 4459.21), and the isotope distribution was as predicted from the sequence (Fig. S1). The amino acid analysis after acid hydrolysis (Table 1) shows a very close correspondence with the expected composition, indicating that the peptide is of the correct sequence and free of contaminating proteins. Five cycles of N-terminal sequencing confirmed the expected residues including the presence of methionine at position 1 (not shown). MS/MS fragment ion analysis confirmed the correct sequence of Aβ(M1–40) (data not shown).

**Table 1:** Amino acid analysis after acid hydrolysis.

Amino acid	Expected composition	Observed composition
Asp + Asn	4	3.9917
Ser	2	2.1648
Glu + Gln	4	4.0643
Gly	6	6.0117
Ala	3	3.0265
Val	5	5.0813
Met	2	1.7661
Ile^a^	2	1.1517
Leu	2	1.9935
Tyr	1	0.96174
Phe	3	2.9287
His	3	2.8426
Lys	2	2.0270
Arg	1	0.99652

a Ile–Ile peptide bonds are known to be inefficiently hydrolyzed.

### Co-expression of Aβ(M1–40) with aminopeptidase

**Fig. 4:**
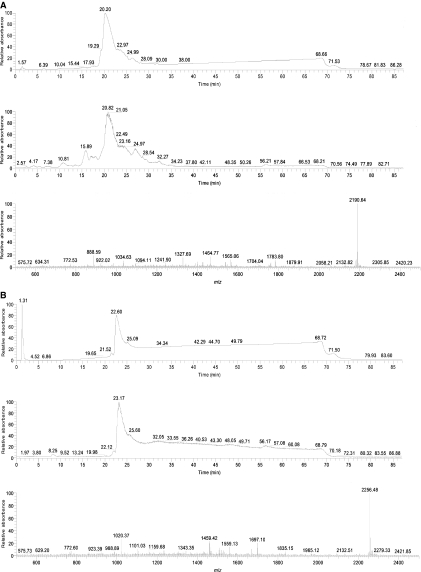
LC-MS analysis of bacterially expressed Aβ(M1–40) (B) confirms the correct molecular mass and indicates that the peptide is of comparable purity to synthetic Aβ(1–40) (A). In each panel, the top panel is the HPLC chromatogram obtained with UV absorption at 214 nm, the middle panel is the corresponding total ion-current after infusion into the mass spectrometer, and the bottom panel is the mass spectrum of the major peak observed.

Mass spectrometric analysis of Aβ(M1–40) and Aβ(M1–42) from several batches very clearly showed the presence of Aβ(M1–40) or Aβ(M1–42), with no indication of any product resulting from spontaneous cleavage of the N-terminal methionine in *E. coli* (Figs 4, S1 and S2). Co-expression of the *E. coli* aminopeptidase methionine aminopeptidase TG (MetAP-TG) [24] and Aβ(M1–40) was therefore attempted, and was found to results in a low yield of Aβ(1–40). Aβ was purified from the cell pellet as described above, and analyzed by MALDI-TOF MS (Fig. S1). Assuming similar ionization of Aβ(M1–40) and Aβ(1–40), we found that less than 20% of Aβ(M1–40) was converted to Aβ(1–40) by this method (Fig. S1), although the expression level of aminopeptidase MetAP-TG was higher than that for Aβ(M1–40) as determined by SDS-PAGE (not shown). MS/MS fragment ion analysis confirmed the correct sequence of the Aβ(1–40) produced by co-expression with aminopeptidase.

### Isolation of monomeric Aβ and kinetic analysis of aggregation

**Fig. 5:**
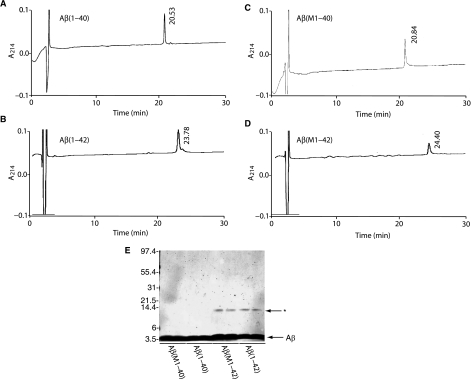
Recombinant and synthetic peptides are highly pure and behave similarly on SDS-PAGE and HPLC. Peptides were isolated by SEC and analyzed by reverse-phase HPLC [(A) Aβ(1–40), (B) Aβ(1–42), (C) Aβ(M1–40) and (D) Aβ(M1–42)] and SDS-PAGE (E). Samples electrophoresed on 10–20% polyacrylamide Tris-tricine gels were detected by silver staining. Monomeric Aβ is indicated by an arrow and an Aβ42 species migrating at approximately 14 kDa is indicated by an arrow and an asterisk.

As aggregation of Aβ peptides is strongly influenced by the presence of structural and chemical impurities, all samples were denatured using 5 m guanidine hydrochloride (GuHCl) in 50 mm Tris-HCl pH 8.0 and subjected to size-exclusion chromatography (SEC) to isolate homogenous monomeric Aβ solutions, as described previously [25]. All four peptides produced a large peak that eluted around 12.5 mL from a Superdex 75 10/30 HR column (data not shown). Further analysis of these peaks by reverse-phase HPLC and SDS-PAGE/silver staining revealed highly pure starting material. In each case, the peptides produced a single peak on HPLC (Fig. 5A–D). The retention times of Aβ(1–40) and Aβ(M1–40) were highly similar and the peaks were typically symmetrical. The retention times and peak shapes for Aβ(1–42) and Aβ(M1–42) were similar to each other, but were distinct from those of the peptides terminating at Val40. The more hydrophobic peptides ending at Ala42 were retained on the column for longer, and produced less symmetrical peaks, as found previously for synthetic peptides [26]. On SDS-PAGE, all four peptides produced a band that migrated at approximately 4 kDa. Given the small molecular mass difference between the peptides ending at Val40 and Ala42, it is not possible to resolve these peptides on standard SDS-PAGE [27]; however, this system is useful to confirm the correct migration of Aβ peptides and their relative purity as assessed by silver staining. In the examples shown, 100 ng of each peptide were loaded per lane, and only a single band was detected in the lanes containing Aβ(1–40) and Aβ(M1–40) (Fig. 5E). In other experiments, 400 ng of peptide were loaded in each well, and very darkly stained broad Aβ bands were detected upon silver staining, but no additional non-Aβ bands were detected. Prior experience indicates that the silver staining protocol used can detect as little as 10 ng of protein [28], thus the present results suggest that SEC-isolated Aβ(1–40) and Aβ(M1–40) are at least 97% pure. In the lanes containing Aβ(1–42) and Aβ(M1–42), there were prominent bands at approximately 4 kDa and faint bands at approximately 14 kDa. The band at approximately 14 kDa is not an impurity as it was present in both the recombinant and synthetic peptides, but probably represents an artifact of SDS-PAGE [29] as it was also detected by Western blotting using anti-Aβ specific antibodies (not shown). Thus, as with the peptides terminating at Val40, Aβ(1–42) and Aβ(M1–42) are also at least 97% pure. Together, these results confirm that our recombinant Aβ(M1–40) and Aβ(M1–42) are at least as pure as the synthetic peptides purified by reverse-phase HPLC, a finding corroborated by LC-MS analysis (Figs 4 and S2).

**Fig. 6:**
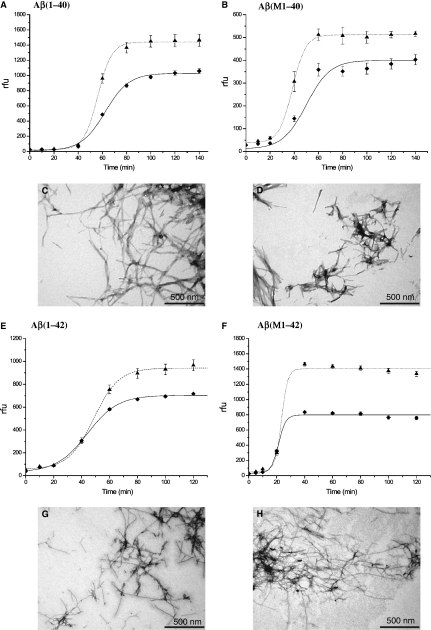
Recombinant and synthetic Aβ peptides exhibit similar amyloid-forming properties. Amyloid fibrils and protofibrils bind to ThT, causing a red shift in the excitation spectrum of this compound. A change in the ThT fluorescence at 480 nm was therefore used to monitored the kinetics of amyloid fibril formation by Aβ(1–40) (A), Aβ(M1–40) (B), Aβ(1–42) (E) and Aβ(M1–42) (F). As Aβ fibrillogenesis is known to be highly concentration-dependent, aggregation was monitored both at 6 μm (diamonds, solid line) and 9 μm (triangles, dashed line). Each data point is the mean of eight replicates ± the standard error; where error bars are not visible, the standard error was smaller than the size of the symbols. In all cases, aggregation exhibits a lag phase, subsequent growth and a final equilibrium phase, and the curves shown were fitted to the data by the Boltzmann equation using origin pro 7.5 software (Northampton, MA, USA). The experiment shown is representative of two identical experiments. For electron microscopy, peptide solutions were incubated at 50 μm for 5 h (Aβ40) or 80 min (Aβ42). Triplicate grids for each peptide at each time point were prepared and viewed. The images shown are for Aβ(1–40) (C), Aβ(M1–40) (D), Aβ(1–42) (G) and Aβ(M1–42) (H). Scale bar = 500 nm.

The fibril-forming properties of Aβ peptides were assessed using a continuous ThT-binding assay and negative-contrast electron microscopy. Aβ(M1–40) and Aβ(M1–42) were compared side by side with Aβ(1–40) and Aβ(1–42) synthesized using standard Fmoc chemistry and isolated by SEC as described above. Thioflavin T binds to Aβ fibrils and protofibrils [30], and has been extensively used to follow the aggregation kinetics of both Aβ and other amyloidogenic proteins [31,32]. At time zero, none of the peptides showed appreciable ThT binding, indicating that the samples were indeed free of structural impurities. After a relatively brief lag phase, ThT binding increased rapidly, quickly reaching maximum values and plateauing thereafter. The rate and extent of aggregation was highly dependent on the concentration of Aβ peptide (Fig. 6A,B,E,F), with Aβ(1–42) and Aβ(M1–42) aggregating faster than Aβ(1–40) and Aβ(M1–40). These aggregation kinetics are typical of many nucleation-dependent polymerization processes, and have been documented in numerous studies on Aβ, in which Aβ42 has been shown to be more amyloidogenic than Aβ40 [32–34]. The morphology of aggregates formed after incubation times when the aggregation had reached a maximum [5 h for Aβ(M1–40) and Aβ(1–40) and 80 min for Aβ(M1–42) and Aβ(1–42)] was assessed by negative contrast electron microscopy, which revealed an abundance of amyloid fibrils in incubates of all four peptides (Fig. 6C,D,G,H). Mats of heavily stained amyloid fibrils were widely distributed over grids containing each of the peptides studied, but electron micrographs of the edges of fibril mats or isolated well-dispersed fibers are presented to show the fibril morphology at high definition. These fibrils vary in length, and can be several micrometers long and with an average diameter of 10.9 nm; no differences in either the length, width or abundance of fibrils were observed between synthetic and recombinant peptides, and the fibrils detected were similar to those previously described [35].

### Toxicity of recombinant and synthetic Aβ peptides

**Fig. 7:**
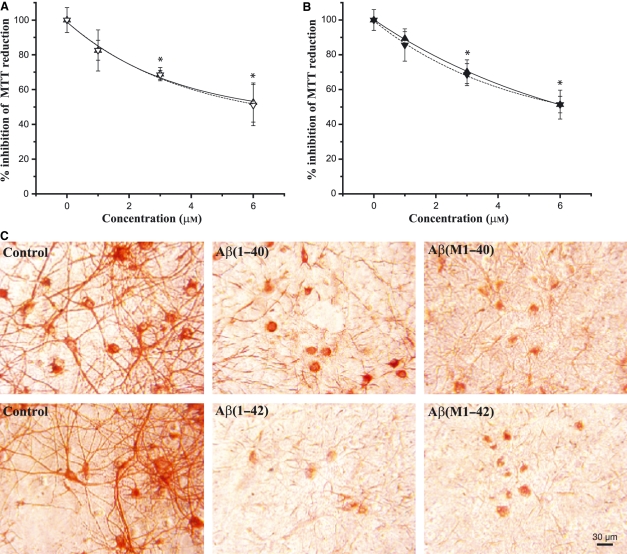
Recombinant Aβ peptides inhibit MTT reduction and cause neuronal loss. Monomeric Aβ peptides were isolated by SEC and incubated at 37 °C with shaking until half-maximal aggregation was observed. Peptides were then diluted into neurobasal medium and incubated with neurons at final concentrations of 1, 3 and 6 μm for 6 h. At the end of this period, MTT was added and cells were incubated for a further 2 h. The results are percentage inhibition of MTT reduction relative to control neurons not treated with peptide, and are the mean of three replicates ± standard deviation. (A) Aβ(1–40) (open triangle) and Aβ(M1–40) (inverted open triangle); (B) Aβ(1–42) (closed triangle) and Αβ(M1–42) (inverted closed triangle). To assess the effect of prolonged incubation with Aβ peptides on cell viability, neurons were incubated with 10 μm Aβ(1–40), Aβ(M1–40), Aβ(1–42) or Αβ(M1–42) for 4 days, fixed and then stained with anti-MAP-2 antibody, viewed by light microscopy using a 40× objective lens and photographed (C). The images shown are at a magnification of approximately 200×.

The precise assembly form(s) of Aβ that cause neuronal compromise are, as yet, ill-defined [36]; thus, rather than attempt to prepare a single Aβ assembly, we deliberately ‘aged’ our peptide preparations until they attained 50% of maximal thioflavin T binding. Using these matched mixed assemblies of recombinant and chemical synthesized peptides, we assessed the effect of both acute and chronic exposure to neurons. For acute experiments, we measured inhibition of MTT reduction, and compared the outcome in cultures that had been treated with synthetic Aβ(1–40) versus recombinant Aβ(M1–40) or synthetic Aβ(1–42) versus recombinant Aβ(M1–42). Firstly, we tested the effect of Aβ peptides on MTT reduction by mature primary rat hippocampal neurons. All four peptides caused a dose-dependent inhibition of MTT reduction that was apparent within 6 h of treatment (Fig. 7A,B), at which time the number and morphology of neurons did not differ either from time zero or from vehicle-treated controls (data not shown). At the three concentrations tested, inhibition of MTT by Aβ(1–40) and Aβ(M1–40) was essentially identical; similarly, the degrees of inhibition caused by Aβ(1–42) and Aβ(M1–42) were indistinguishable at each concentration studied. Moreover, the extent of MTT inhibition was not significantly different for peptides ending at residues 40 and 42, with approximately 50% inhibition at 6 μm for all four peptides. Longer-term treatment of neurons with the same peptides caused neuritic degeneration and loss of neurons (Fig. 7C), with a similar loss evident for all peptides.

## Discussion

Because extensive evidence supports a crucial role for Aβ in Alzheimer’s disease pathogenesis, there is huge interest in understanding the structural and biological properties of this molecule [13–16]. Using chemically synthesized Aβ peptides, substantial progress has been made in understanding of the aggregation and toxic properties of Aβ assemblies [17,37]. However, given that chemically synthesized Aβ peptides are expensive to purchase and/or make, this has curtailed the extent of experiments, and may have deterred new investigators from studying Aβ, or forced others to study small irrelevant fragments (e.g. Aβ25-35) rather than the full-length Aβ sequence. Thus, we set ourselves the goal of developing a facile inexpensive procedure for the production of recombinant Aβ peptides. In addition to being more cost-effective than production of synthetic peptides, a bacterial expression system allows isotope labeling, which is essential for high-resolution structural analysis of Aβ using NMR spectroscopy and allows use of high specific activity radiotracers to study Aβ uptake, transport and clearance. Moreover, a recombinant system should also allow the generation of Aβ peptides with design or disease-associated amino acid substitutions, and we have produced some such peptides in this study. Importantly, the protocol described for expression and purification of Aβ(M1–40) and Aβ(M1–42) is inexpensive, relatively rapid and only utilizes rudimentary equipment that is available in most biochemistry laboratories.

Recombinant expression in *E. coli* of human proteins smaller than about 50 residues is often hampered by proteolytic degradation of unstructured proteins/peptides; therefore small entities are commonly expressed fused to a larger protein to prevent degradation. A common drawback of such approaches is the cost of the affinity resins used to isolate the fusion protein and the proteases required to liberate the protein of interest from the fusion protein. Such considerations lead to practical obstacles in terms of scale-up of the purification and consequently the amount of pure peptide that can be produced at reasonable cost. Thus we decided to express the Aβ(M1–40) and Aβ(M1–42) peptides without fusion to another protein. The rationale behind this approach was simple. Aβ peptides show a strong propensity to aggregate, with aggregation proceeding rapidly at high peptide concentrations [33,38,39], thus high-level expression of Aβ peptides should lead to aggregation and formation of inclusion bodies, and that Aβ would be less susceptible to degradation in this form. Moreover, the formation of inclusion bodies enables high-level expression because the peptide is cleared from the bacterial cytosol and hence does not interfere with any essential functions. In addition, proteins deposited in inclusion bodies contain fewer *E. coli* proteins, thus simplifying purification.

The purification protocol that we have developed is quick and efficient. The peptide is produced at high yield in *E. coli* as inclusion bodies, which are washed by sonication, solubilized in urea, purified by anion-exchange chromatography in batch format, and finally any aggregates removed using SEC. The advantage of this protocol is that it relies on affordable tools and can be scaled up to any production size. The batch mode has the additional advantage of avoiding precipitation. In batch mode, the peptide is spread out over the entire resin and is eluted from the resin into buffer in relatively dilute form, which is controlled by the amount of resin and buffer volume used. In column mode, the peptide becomes more concentrated and the yield of eluted monomer is much reduced compared to batch mode due to its aggregation tendency. In column mode, the peptide is bound in a concentrated manner at the top of the column, or, if bound to the resin prior to packing the column, the salt gradient concentrates the peptide on its way out of the column. The molecular mass fractionation in centrifugal devices leads to smaller losses than gel filtration due to more rapid handling using the devices and loss of peptide on the column resin. The only detriment of the peptides produced here is the fact that they contain an exogenous N-terminal methionine. However, the presence of this methionine is not insurmountable, and we have found that co-expression of the *E. coli* aminopeptidase MetAP-TG [24] and Aβ(M1–40) results in a low-yield production of Aβ(1–40); however, separation of Aβ(1–40) and Aβ(M1–40) requires an additional HPLC step and substantially increases the cost and complexity of production.

Additionally, the presence of the exogenous N-terminal methionine does not affect the fibrillation kinetics or morphology of the fibrils formed by Aβ(M1–40) or Aβ(M1–42). Thus such peptides should prove useful in high-throughput screens designed to identify molecules or conditions that modulate Aβ fibrillogenesis. Moreover, these peptides have indistinguishable effects on hippocampal neurons, causing inhibition of MTT reduction within 6 h of treatment and neuritic degeneration and cell loss upon prolonged treatment. Importantly, these results indicated that an N-terminal aspartate is not necessary for neurotoxicity. An additional advantage of the N-terminal methionine is the fact that this residue will not be easily seen in NMR spectra relying on amide protons as the N-terminal amine protons are likely to exchange rapidly with water [40]; thus the presence of the N-terminal methionine may enable detection of Asp1 that would otherwise be invisible in ^1^H^15^N-HSQC spectra. Therefore, the presence of the N-terminal methionine will allow a broader coverage of structural assignments to the N-terminus of Aβ. Moreover, the procedures described here are also suitable for expression and purification of mutant versions of Alzheimer’s disease-associated amyloid β-peptides. In this work, we have evaluated this capacity by producing and cloning genes for familial mutants in the 19–23 region of Aβ(Μ1–40) and Aβ(Μ1–42), and expressing and purifying the peptides. The procedure is of course not limited to the peptide variants produced in this work (F19P, A21G, E22G, E22K, E22Q and D23N), and the availability of a rapid and simple expression and purification protocol will facilitate large-scale investigations of the molecular determinants of aggregation and fibrillation. Given the intense interest in Aβ, significant attempts to produce pure recombinant Aβ have also been made by several other groups, but most of these have relied on the generation of Aβ fusions. Perhaps the best of these was reported by Lee *et al.* using a system in which Aβ was fused to ubiquitin. This protocol relies on the use of Ni-NTA affinity chromatography for purification and subsequent liberation of Aβ by digestion using yeast ubiquitin hydrolase, but the authors did not provide data on the purity of the end product [21]. Similarly, Wieschan *et al.* also employed a fusion strategy, Ni-NTA affinity chromatography and digestion with thrombin [22]. Zhang *et al.* also used a fusion strategy coupled with GSH affinity chromatography and subsequent thrombin cleavage [41]. The use of thrombin significantly increases the cost of the purification, and the requirement for HPLC increases the length and complexity of the purification procedure. Moreover, as with the studies by Lee *et al.* [21] and Subramanian and Shree [42], there was no rigorous assessment of the purity of the product or the correctness of the sequence. In contrast, the purification protocol that we have developed is quick and efficient, and leads to the production of highly pure Aβ peptides with the anticipated molecular mass, amino acid composition, correct primary sequence and appropriate biophysical and neurotoxic characteristics. In short, the protocol described has the potential to facilitate a massive increase in the number and extent of studies aimed at better understanding the molecular details of Aβ oligomerization and aggregation.

## Experimental procedures

Unless otherwise stated, all chemicals were purchased from Sigma-Aldrich (St Louis, MO, USA) and were of the highest purity available. Synthetic peptides Aβ(1–40) and Aβ(1–42) were synthesized in the W. M. Keck Foundation Biotechnology Resource Laboratory (Yale University, New Haven, CT, USA), and purified using reverse-phase HPLC. For both synthetic and recombinant Aβ peptides, the correct mass was confirmed by MALDI-TOF MS and LC-MS.

### PCR and cloning procedure

Synthetic genes for Aβ(M1–40) and Aβ(M1–42) were designed using *E. coli*-favored codons preceded by an ATG initiation codon (Fig. 1). The requirement for a start codon adds a methionine residue at the N-terminus; hence, the peptides expressed here are referred to as Aβ(M1–40) and Aβ(M1–42).

The synthetic gene for Aβ(M1–40) was produced by PCR using Pfusion DNA polymerase (Finnzymes, Espoo, Finland) according to the manufacturer’s guidelines and using the following primers: Aβa, 5′-ATGGACGCTGAATTCCGTCACGACTCTGGTTACGAAGTTCACCACCAGAAGCTGGTG-3′; Aβb, 5′-GTTCACCACCAGAAGCTGGTGTTCTTCGCTGAAGACGTGGGTTCTAACAAGGGTGCT-3′; Aβc, 5′-CACAACGCCACCAACCATCAGACCGATGATAGCACCCTTGTTAGAACCCAC-3′; Aβstart, 5′-GCGTAGGGTCGACATATGGACGCTGAATTCCGTCACG-3′; Aβstop, 5′-CCTGCCGAGCTCCTATTACACAACGCCACCAACCATCAG-3′.

The PCR solution was prepared in the buffer supplied with the enzyme, and contained Aβa, Aβb and Aβc at 40 nm each, and the start and stop primers Aβstart and Aβstop at 600 nm each, and 200 μm each of dATP, dCTP, dGTP and dTTP. The product was separated from primers by agarose gel electrophoresis (2% gel). The full-length gene was cut out from the gel, purified using a GFX PCR and gel band purification kit (GE Healthcare, Chalfont St Giles, UK). The gene was digested with *Nde*I and *Sac*I restriction enzymes and subjected to a second agarose gel electrophoresis (2% gel), and the cleaved product was purified using the GFX PCR and gel band purification kit. The purified cut gene was ligated into PetSac vector (a modified from of Pet3a with *Nde*I and *Sac*I cloning sites [43]) that had been previously cleaved by *Nde*I and *Sac*I, and used to transformed Ca^2+^-competent *E. coli* cells (ER2566) by heat shock. The transformed cells were spread on LB agar plates containing ampicillin (50 mg·L^−1^), single colonies were picked for 2 mL overnight cultures in LB medium containing ampicillin (50 mg·L^−1^), and plasmids were prepared using a GFX plasmid purification kit (GE Healthcare) and sequenced.

The gene for Aβ(Μ1–42) was then produced by PCR using the primers Aβstart and Aβ42stop (5′-CCTGCCGAGCTCCTATTAAGCGATCACAACGCCACCAACCATCAG-3′) and a sequence-verified plasmid carrying the Aβ(Μ1–40) gene. This adds Ile41 and Ala42 to the peptide sequence. The PCR product corresponding to the full-length Aβ(Μ1–42) gene was purified as above and ligated into PetSac. In our PCR design, regions encompassing residues 1–6, 12–18, 24–30 and 34–40 were used as primer annealing sites, and the following codons in these regions were altered to achieve more stable duplexes and/or avoid repeat of similar sequences (K16, AAA→AAG; V24, GTT→GTG; K28, AAA→AAG; G38, GGT→GGC; V40, GTT→GTG). Residues 21–23 are mutated in several Alzheimer’s-like familial disorders [44–48]. In our design, residues 19–23 are therefore uniquely encompassed by the middle primer, such that only one additional primer is required for the production of synthetic genes bearing Alzheimer’s disease-associated point mutants.

### Bacterial expression

Sequence-verified plasmids from wild-type and each mutant were transformed into Ca^2+^-competent *E. coli* cells (BL21 DE3 PLysS Star) by heat shock and spread on LB agar plates containing ampicillin (50 mg·L^−1^) and chloramphenicol (30 mg·L^−1^). Single colonies were used to inoculate 50 mL overnight cultures in LB medium with ampicillin (50 mg·L^−1^) and chloramphenicol (30 mg·L^−1^). The next morning, 5 mL of overnight culture was transferred to 500 mL day culture (LB medium with 50 mg·L^−1^ ampicillin and 30 mg·L^−1^ chloramphenicol). When the density of cells was sufficient to produce an attenuance at 600 nm (*D*_600 nm_) of approximately 0.6, protein expression was induced by addition of isopropyl thio-β-d-galactoside. The cells were harvested between 3 and 4 h after induction, dispensed in Millipore (Carrigtwohill, Cork, Republic of Ireland) H_2_O (12–25 mL H_2_O per liter culture), and frozen.

To assay and optimize expression levels, test samples of 1 mL cultures were collected for each transformed bacterial culture at various temperatures (30, 37 and 41 °C) and at various times (1, 2, 3, 4, 5 or 6 h) after induction, and using seven different isopropyl thio-β-d-galactoside concentrations ranging from 0.1 to 2.0 mm for induction. The cell suspension was centrifuged at 5400 ***g*** and 4 °C for 15 min, the cell pellet was resuspended in H_2_O (100 μL) and centrifuged again, after which the supernatant was collected and the pellet dissolved in 8 m urea (100 μL). Both the supernatant and urea-solubilized pellet were then analyzed by agarose gel electrophoresis at pH 8.4 and by SDS-PAGE.

### Sonication

The frozen cell pellet from a 4.5 L culture was thawed, sonicated in a total of 100 mL 10 mm Tris/HCl pH 8.0, 1 mm EDTA, for 2 min on ice (1/2 horn, 50% duty cycle), and centrifuged for 10 min at 18 000 ***g***. The supernatant (S1 in Fig. 2) was removed, and the pellet was resuspended twice in 100 mL 10 mm Tris/HCl pH 8.0, 1 mm EDTA, sonicated and centrifuged as above. The third supernatant was removed, and the pellet was resuspended in 50 mL 8 m urea, 10 mm Tris/HCl pH 8.0, 1 mm EDTA, and sonicated as above, resulting in a clear solution. To minimize carbamylation of Aβ, fresh solutions of ice-cold, deionized ACS grade urea were used, and the duration of exposure to urea was limited to less than 12 h.

### Purification of Aβ(M1–40) and Aβ(M1–42)

The procedures described here are for 50 mL of urea-solubilized inclusion bodies originating from 4.5 L of culture, but this process can be scaled proportionally for other amounts. The urea-solubilized inclusion bodies (50 mL) were diluted with 150 mL of 10 mm Tris/HCl pH 8.0 containing 1 mm EDTA (buffer A), added to 50 mL DEAE-cellulose equilibrated in buffer A, and gently agitated for 20 min. The slurry was then applied to a Büchner funnel with filter paper on a vacuum glass bottle [alternatively, a Nalgene (Lima, OH, USA) 0.45 μm filter on a vacuum bottle can be used]. Subsequently, the resin was washed with buffer A (50 mL), followed by stepwise elution using 50 mL aliquots of buffer A with 50, 75, 100, 125, 150, 200, 250, 300 and 500 mm NaCl, respectively. Each aliquot was incubated with the resin for 5 min before collection under vacuum. Eluates were analyzed by SDS-PAGE and agarose gel electrophoresis, and fractions with highly pure Aβ were pooled and fractionated by centrifugation through a 30 kDa molecular mass cut-off filter. The washing and elution processes can also be performed as follows: the resin is washed with 50 mL buffer A, and then with 50 mL buffer A with 25 mm NaCl followed by three or four 50 mL aliquots of buffer A with 125 mm NaCl. Using SDS-PAGE, the peptide is then found in the first two (or first three) 125 mm aliquots, which are combined and used for centrifugal filtration.

### Ion-exchange chromatography in column mode

Urea-solubilized inclusion bodies (25 mL originating from 2.2 L of bacterial cell culture) were diluted with 150 mL of buffer A and applied to a 50 mL DEAE-cellulose column equilibrated in buffer A. The column was washed with 50 mL buffer A, followed by elution using a linear gradient from 0–300 mm NaCl with a total gradient volume of 500 mL. Fractions were analyzed by electrophoresis on 10–20% polyacrylamide Tris-tricine gels and 1% agarose gels. In a second set of experiments, the column was equilibrated in buffer A containing 8 m urea, and the sample was eluted with a gradient of 0–300 mm NaCl in buffer A containing 8 m urea.

### Mass spectrometry, amino acid analysis and sequencing

Amino acid analysis was performed at the Amino Acid Analysis Center, University of Uppsala, Sweden. Sequence analysis was performed using an Applied Biosystems Procise 492 cLC sequenator (Applied Biosystems, Framingham, MA, USA) employing standard Edman chemistry, and MS analysis was undertaken using an LCQDECA LC/MS system (ThermoFinnigan, San Jose, CA, USA). The MS system consisted of a Surveyor HPLC system with a diphenyl 150 × 1.0 mm column (Grace Vydac, Palo Alto, CA, USA) interfaced to an LCQ-DECA electrospray ionization/ion trap mass spectrometer, and eluted using an acetonitrile/trifluoroacetic acid gradient. MALDI-TOF mass spectrometry was performed using a 4700 proteomics analyzer (Applied Biosystems). Samples were dispensed onto a MALDI sample support, and allowed to air-dry prior to addition of matrix solution (4-hydroxy α-cyano cinnamic acid in 50% acetonitrile, 0.1% trifluoroacetic acid, 25 mm citric acid). All analyses were performed in positive reflector mode, collecting data from approximately 3000 and 5000 single laser shots for MS and MS/MS analyses, respectively.

### Preparation of aggregate-free monomer for fibrillation assays

For fibrillation assays, it is essential to start with a uniform monomeric peptide sample. Solutions of monomeric Aβ were prepared by dissolving lyophilized peptides in 5 m GuHCl, Tris/HCl pH 8.0 at a concentration of approximately 1 mg·mL^−1^, and isolating monomers using SEC. Aβ solutions were chromatographed on a Superdex 75 10/300 GL column using an ÄKTA purifier (GE Healthcare), and eluted at 0.8 mL·min^−1^ using 50 mm ammonium acetate, pH 8.5. Fractions (0.5 mL) were collected, peak fractions pooled, and the concentration of peptide determined by absorbance at 275 nm using ε_275_ = 1400 m^−1^ cm^−1^.

### Assessment of aggregation using thioflavin T binding and electron microscopy

The kinetics of fibril formation was determined using a continuous ThT assay [49]. Solutions of Aβ isolated by SEC were diluted to concentrations of 36 or 24 μm using 50 mm ammonium acetate, pH 8.5. Peptides were then incubated in a 96-well black fluorescence plate at a final concentration of 6 or 9 μm in the presence of 10 μm ThT at 37 °C, and shaken at 700 r.p.m. using a VorTemp 56™ incubator/shaker with an orbit of 3 mm (Labnet International, Windsor, UK). Measurements were made at regular intervals using a SpectraMax M2 microplate reader (Molecular Devices, Sunnyvale, CA, USA) with excitation and emission at 440 and 480 nm, respectively. Each experimental point is the mean of the fluorescence signal of at least eight wells containing aliquots of the same solution. The morphology of Aβ aggregates formed from solutions incubated as above but in the absence of ThT and at a concentration of 50 μm was assessed by negative-contrast electron microscopy as described previously [25]. Briefly, samples were applied to a carbon-coated formvar grid, left for 1 min, fixed with glutaraldehye, wicked dry with filter paper, and 2% uranyl acetate was added and the mixture was incubated for 2 min. The grid was wicked dry and allowed to air dry for 10 min. Samples were stored in a sealed container and viewed under a Tecani G2 BIOTWIN electron transmission microscope operated at 120 V. All reagents were supplied by Electron Microscopy Sciences (Hatfield, PA, USA).

### Assessement of SEC-isolated peptides by HPLC and SDS-PAGE

Samples (100 μL) of peptides isolated by SEC were injected on to a CN capcell column (4.6 mm × 25 cm) (Shiseido Fine Chemicals, Toyko, Japan) using a Varian Pro Star 410 autosampler (Varian Inc., Palo Alto, CA, USA), and eluted at 1.5 mL·min^−1^ with a 14–49% acetonitrile gradient using a Varian Pro Star HPLC system fitted with a photodiode array detector. For SDS-PAGE, samples (10 μL) were mixed with 2× sample buffer, and immediately electrophoresed on 10–20% polyacrylamide Tris-tricine gels. Proteins were stained with silver as described previously [28].

### Primary culture

Primary hippocampal neuronal cultures were prepared as described previously [30] with minor modifications. Briefly, primary hippocampal cultures were generated from embryonic day 18 Wistar rats. Hippocampi were dissected out in Hanks’ balanced salt solution buffered with HEPES, and dissociated using papain. Cells were plated at 6 × 10^4^ cells on 48-well dishes pre-coated with poly-d-lysine (50 μg·mL^−1^) and maintained in neurobasal medium containing 2 mm glutamine and B27 supplement without antioxidants. Half the medium was exchanged every 3 days. All media reagents were purchased from Invitrogen (Dun Laoghaire, Republic of Ireland).

### Preparation of peptide for cell treatment

Lyophilized peptides were resuspended and incubated for a minimum of 2 h in 5 m GuHCl, pH 8.0. Thereafter, samples were injected onto a Superdex 75 column HR 10/30 column (Amersham Biosciences, Amersham, UK), and eluted with 10.9 mm HEPES pH 7.4 at a flow rate of 0.8 mL·min^−1^. Peak fractions were then examined for absorbance at 275 nm, and the concentration of Aβ was calculated. Fractions containing monomeric peptide were diluted such that all peptides were of equal concentration. To induce peptide aggregation, samples were incubated at 37 °C and shaken at 700 r.p.m. using a VorTemp 56™ incubator/shaker with an orbit of 3 mm (Labnet International) until 50% of the maximal thioflavin T fluorescence had been achieved; maximal aggregation was taken as the mean plateau fluorescent signal. Peptides were then diluted with 2× neurobasal medium, and 50% of the medium of each well was replaced with an equal volume of neurobasal medium containing either Aβ(1–40), Aβ(M1–40), Aβ(1–42) or Aβ(M1–42) (1, 3 or 6 μm, final concentration) and incubated for 6 h. Cell-mediated reduction of 3-(4,5-dimethylthiazol-2-yl)-2,5-diphenyltetrazolium bromide (MTT) was assessed as described previously [30]. Briefly, following incubation with peptides, 2.5 mg·mL^−1^ MTT (25 μL) was added to each well, and incubation was continued for a further 2 h. Cells were then solubilized in 250 μL of 20% w/v SDS in 50% v/v *N,N′*-dimethylformamide, 25 mm HCl, 2% v/v glacial acetic acid, pH 4.7, and levels of reduced MTT were determined by measuring the difference in absorbance at 570 and 650 nm using a Molecular Devices Spectramax M2 microplate reader.

In a separate series of experiments, neurons were incubated for 4 days with each of the peptides (10 μm), and cells were fixed and used for immunocytochemical analyses.

### Immunocytochemistry

Neurons were fixed in 4% paraformaldehyde for 20 min at room temperature, and cells were stained for microtubule-associated protein-2 (MAP-2) using a Vectastain kit (Vector Laboratories, Peterborough, UK). Staining was performed according to the manufacturer’s instructions. Briefly, endogenous peroxidases were blocked in 0.3% H_2_O_2_, rinsed in NaCl/P_i_ and incubated in blocking solution for 20 min (Vectastain). Neurons were then incubated with mouse monoclonal anti-MAP-2 (Sigma, Poole, UK) diluted 1 : 2000 in blocking solution for 30 min. Cells were rinsed in NaCl/P_i_ several times and incubated in blocking serum containing anti-mouse IgG (Vectastain) for a further 30 min. Staining was developed by incubation of cells with Vectastain ABC reagent for 30 min, followed by incubation with substrate solution until colour had developed. Cells were visualized by light-phase contrast microscopy using a 40× objective lens, and captured using an SP-500 UZ digital compact camera (Olympus, Watford, UK).

### Co-expression with Met aminopeptidase

Plasmids encoding MetAP-TG (a mutated form of Met aminopeptidase that can cleave N-terminal Met when the second residues is charged [24]) and Aβ were electroporated into *E. coli* cells (BL21 DE3 PLysS Star) and spread on LB plates with ampicillin, kanamycin and chloramphenicol. Single colonies were picked for cultivation in liquid culture as described for Aβ alone, except that the medium contained 50 mg·L^−1^ ampicillin, 100 mg·L^−1^ kanamycin and 30 mg·L^−1^ chloramphenicol.

## Abbreviations

Aβ: amyloid β-peptide
GuHCL: guanidine hydrochlorise
MetAP-TG: methionine aminopeptidase TG
MTT: 3-(4,5-dimethylthiazol-2-yl)-2,5-diphenyltetrazolium bromide
SEC: size-exclusion chromatography
ThT: thioflavin T

## Supporting information

The following supplementary material is available:

**Fig. S1.** MS analysis of bacterially expressed Aβ(M1–40).

**Fig. S2.** LC-MS analysis of bacterially expressed Aβ(M1–42) con.rms the correct molecular mass and indicates that the peptide is of comparable purity to synthetic Aβ(1–42).

This supplementary material can be found in the online version of this article.
