# Supplementary material for: A facile method for expression and purification of the Alzheimer’s disease-associated amyloid β-peptide
Source: FEBS J. 2009 Mar;276(5):1266–81. doi: 10.1111/j.1742-4658.2008.06862.x (PMC2702495; doi:10.1111/j.1742-4658.2008.06862.x)

Supplementary Figure S1. Mass Spectrometric analysis of bacterially expressed Aβ(M1-40).

MALDI-TOF mass spectra of (A) intact Aβ(M1-40) peptide and (B) Aβ(M1-40) peptide co-expressed with Met aminopeptidase. (C) Magnification of the m/z range highlighted in (B) with the signal at m/z 4328.16 representing the N-terminally processed Aβ(1-40) peptide.

Supplemental Figure S2. Liquid chromatography-mass spectrometric analysis of bacterially expressed Aβ(M1-42) (panel B) confirms the correct molecular weight and indicates the peptide is of comparable purity to synthetic Aβ(1-42) (panel A).

In each panel, the top figure is the HPLC chromatogram obtained with UV absorption at 214 nm and the bottom figure is the mass spectrum of the major peak observed.

Walsh et al. Suppl Fig. 1

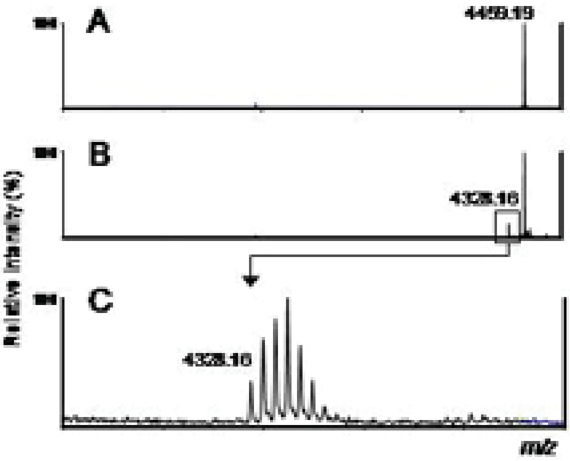

Walsh et al. Suppl Fig. 2

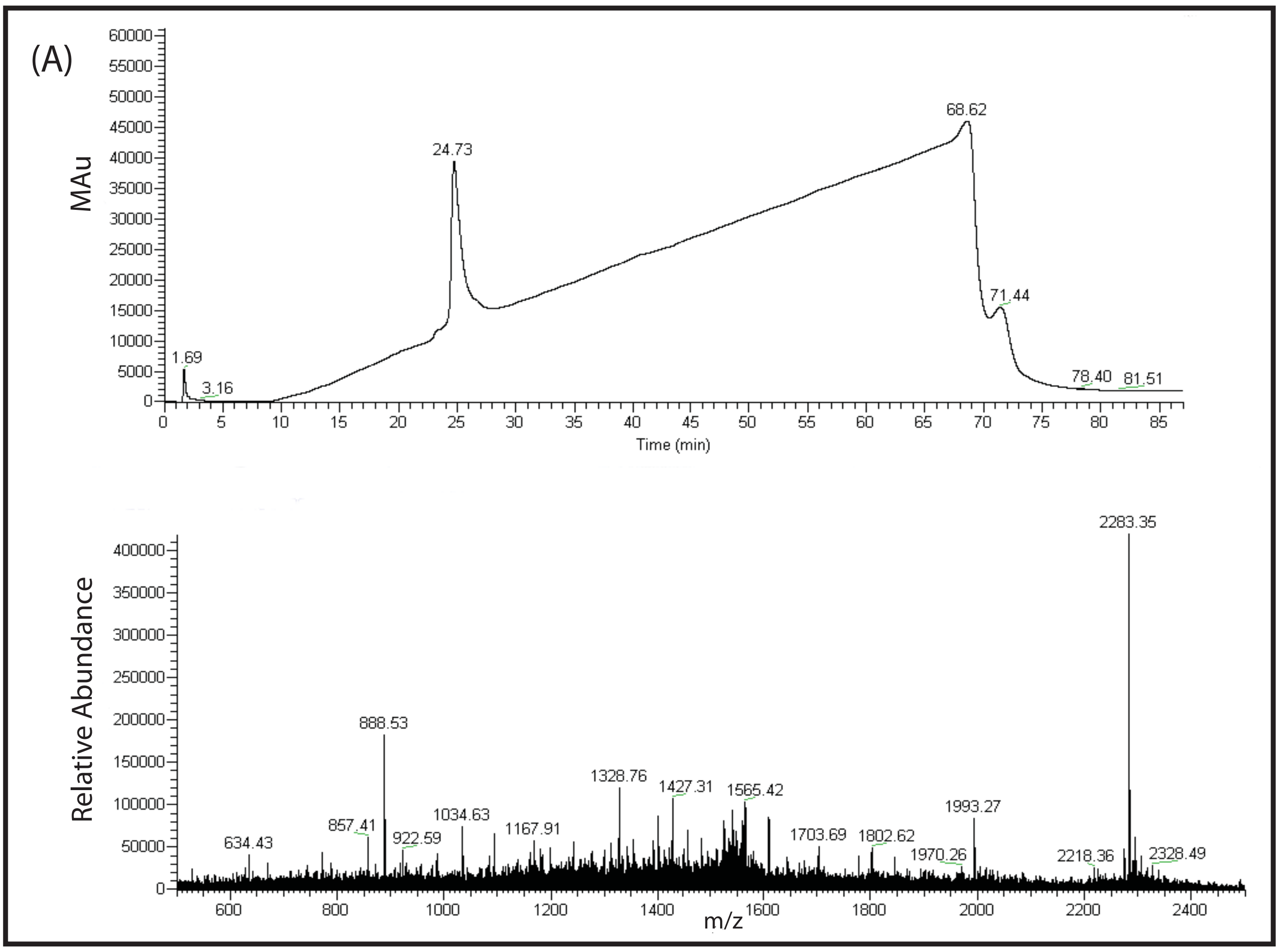

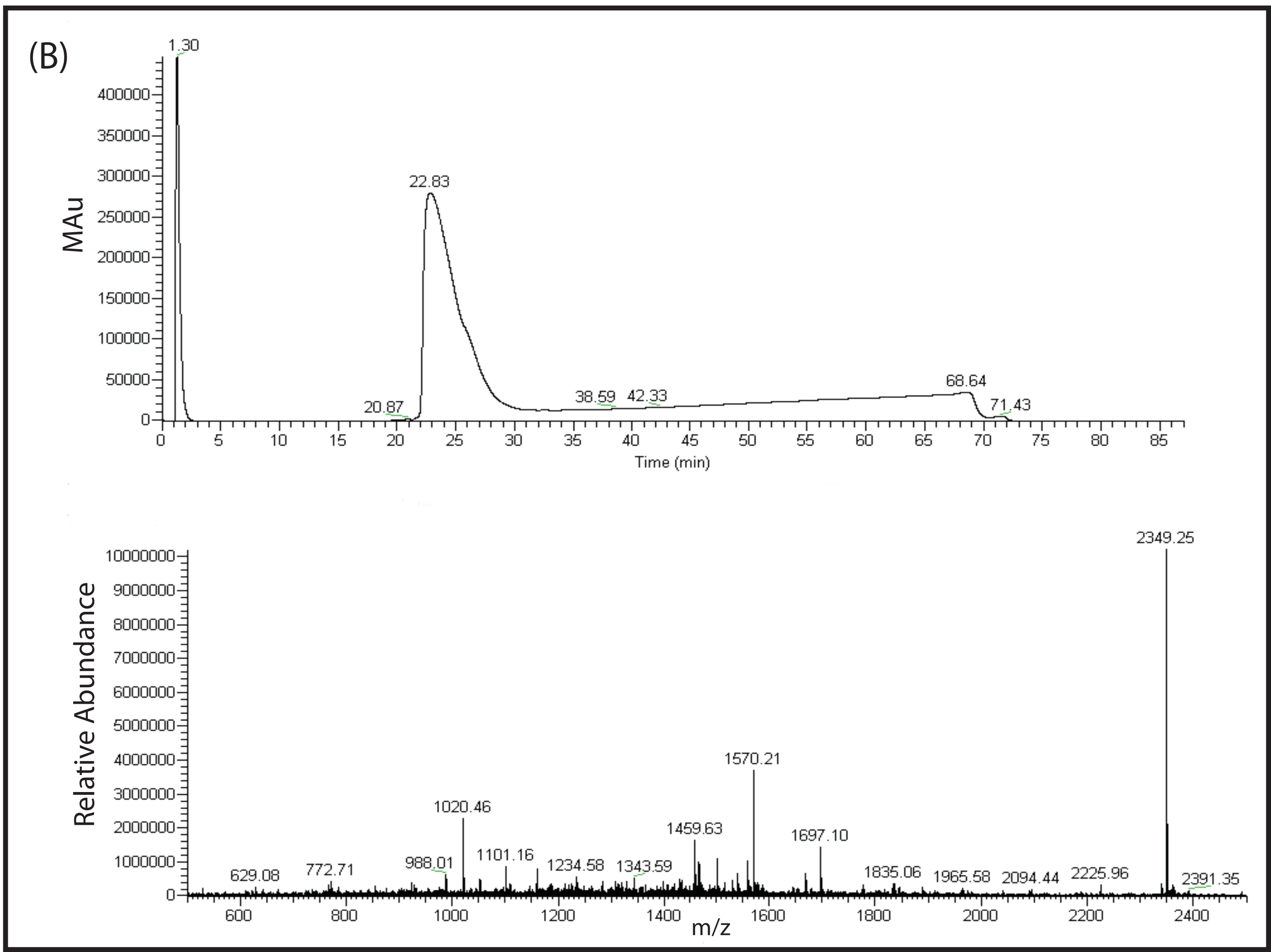

Supplement: Supplementary file 1 [file ejb0276-1266-SD1.pdf]
